# Digital spatial profiling identifies molecular changes involved in development of colitis-associated colorectal cancer

**DOI:** 10.3389/fonc.2024.1247106

**Published:** 2024-03-04

**Authors:** Tamara Glyn, Sarah Williams, Martin Whitehead, Tim Eglinton, Nicholas West, Rachel V. Purcell

**Affiliations:** ^1^ Department of Surgery and Critical Care, University of Otago, Christchurch, New Zealand; ^2^ Griffith Health, Griffith University, Gold Coast, QLD, Australia; ^3^ Department of Anatomical Pathology, Te Whatu Ora Waitaha, Christchurch, New Zealand

**Keywords:** colorectal (colon) cancer, IBD - inflammatory bowel disease, inflammation, spatial gene expression, colitis

## Abstract

**Objective:**

Chronic colonic inflammation seen in inflammatory bowel disease (IBD) is a risk factor for colorectal cancer (CRC). Colitis-associated cancers (CAC) are molecularly different from sporadic CRC. This study aimed to evaluate spatially defined molecular changes associated with neoplastic progression to identify mechanisms of action and potential biomarkers for prognostication.

**Design:**

IBD patients who had undergone colectomy for treatment of their IBD or dysplasia were identified from an institutional database. Formalin-fixed paraffin embedded samples from areas of normal, inflamed, dysplastic and adenocarcinoma tissue were identified for digital spatial profiling using the Nanostring GeoMx™ Cancer Transcriptome Atlas. RNA expression and quantification of 1812 genes was measured and analysed in a spatial context to compare differences in gene expression.

**Results:**

Sixteen patients were included, nine patients had CAC, two had dysplasia only and five had colitis only. Significant, step-wise differences in gene expression were seen between tissue types, mainly involving progressive over-expression of collagen genes associated with stromal remodelling. Similarly, MYC over-expression was associated with neoplastic progression. Comparison of normal and inflamed tissue from patients who progressed to those who did not also showed significant differences in immune-related genes, including under-expression of thte chemokines CCL18, CCL25 and IL-R7, as well as CD3, CD6 and lysozyme. The known oncogene CD24 was significantly overexpressed.

**Conclusion:**

Both tissue types and patient groups are molecularly distinguishable on the basis of their gene expression patterns. Further prospective work is necessary to confirm these differences and establish their clinical significance and potential utility as biomarkers.

## Introduction

The term inflammatory bowel disease (IBD) encompasses ulcerative colitis (UC) and Crohn’s disease (CD), and describes an immune mediated inflammation of the gut. UC typically presents as mucosally based proctitis extending proximally in a contiguous fashion and involving the colon, whereas CD may manifest at any location in the gastrointestinal tract, causing transmural inflammation and ‘skip lesions’ with regions of macroscopically ‘normal’ bowel intervening. Chronic colonic inflammation is associated with an increased risk of colorectal cancer (CRC); this is proportional to the extent, degree and duration of inflammation (1–3). There is some evidence to suggest that colitis associated CRC (CAC) are more likely to present with higher histologic grade of CRC and have worse overall survival (4–6). CRC is a significant contributor to the overall morbidity and mortality in IBD, with 14% of deaths attributed to CRC (7, 8).

Surveillance colonoscopy is recommended for patients with significant chronic colonic inflammation based on clinically based, individualised risk stratification (9), however, repeated colonoscopy is expensive, invasive and poor at risk-prediction, resulting in both over- and under-treatment. This is in part due to a lack of ability to distinguish those who will progress from inflammation or low-grade dysplasia (LGD) to invasive CRC (progressors), and those ‘non-progressors’ whose disease will follow a more indolent course (10–12). Identifying and validating potential biomarkers that reliably predict which patients will progress to invasive disease would allow a more tailored surveillance and management plan to be devised for individuals and be critical to clinical decision making.

The identification of biomarkers requires a thorough understanding of the pathogenesis and molecular changes underpinning colitis-associated CRC (CAC). Several pathways and mutations identified in sporadic CRC are seen in CAC, however, there are significant differences that imply a distinct inflammatory-mediated process driving CAC (13).

To date, no biomarkers have been identified that are sufficiently consistent, reliable and clinically relevant to have progressed into clinical practice. Molecular profiling of CAC has suggested alterations in gene expression in pathways related to i) transcription and DNA repair; ii) cell cycle and growth; iii) cellular metabolism; iv) cellular communication and v) signal transduction (14). Loss of gene expression, especially of immune response proteins, is associated with neoplastic progression (14).

Most techniques used to quantify gene expression, so far, have been spatially agnostic, limiting the interpretability of the findings. More recently, digital spatial profiling (DSP) techniques have been developed that allow the quantification and localisation of gene expression, facilitating a better understanding of the significance of these changes within the tissue architecture (15). This is particularly useful in the context of our current paradigm of CAC development, which suggests a complex interaction between the mucosa, its microbiome and the immune microenvironment (16).

The aim of this study was to utilise DSP to characterise the molecular and cellular changes associated with transformation of normal and inflamed tissue to dysplasia and invasive CAC, in order to derive potential biomarkers to distinguish risk of progression at an earlier stage. The secondary objective was to investigate changes in the immune-cell populations associated with CAC, in order to better understand the role of the immune microenvironment in malignant transformation.

## Materials and methods

### Patient selection and ethics

Adult patients (>18 years) who had undergone resection of colonic tissue for IBD-related indications were eligible for inclusion. Inclusion criteria included a histologically confirmed diagnosis of IBD, availability of tissue of adequate quality for analysis, availability of sufficient clinical and demographic detail and the capacity to provide consent. Exclusion criteria included age under 18 years, lack of tissue, lack of confirmed diagnosis of IBD, unable or unwilling to provide consent.

Patients were identified from review of the prospectively maintained Canterbury Inflammatory Bowel Disease Project (CIBDP) and an institutional operative database. Eligibility was then confirmed through review of clinical and pathologic details. Patients were contacted and written consent obtained.

Ethics approval was obtained from the Northern B Health and Disability Ethics committee (19/NTB/144) and locality authorisation was obtained through Canterbury District Health Board (CDHB) Research Office (RO20162).

### Patient and public involvement

As part of the ethics process in New Zealand, the local Māori ethics committee has input into the design of the study. The intention is to ensure equity and community issues are addressed in an appropriate and sensitive manner. In this study, this influenced management of data and tissue. Patient groups were not specifically consulted, however individual informed consent was obtained and those patients were asked if they would like to receive a summary of the results of the study; those that chose to will receive this.

### Morphology staining and region of interest identification

#### Digital spatial profiling

**Figure 1:**
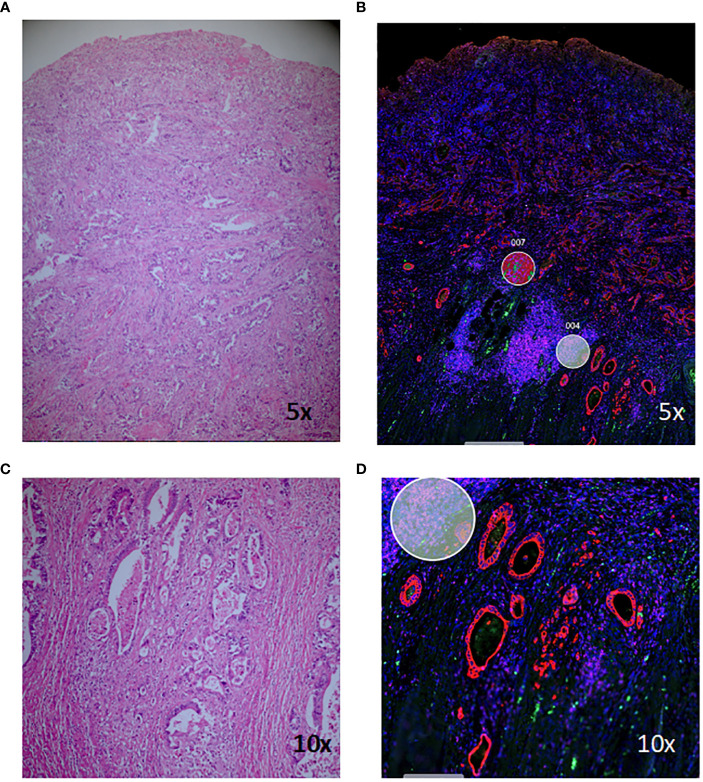
Haematoxylin and eosin (H+E) and morphology-marker stained sections for digital spatial analysis. Staining of a representative section of colitis-associated colorectal cancer **(A, C)** and corresponding morphology marker-stained sections **(B, D)**. Red, PanCK; green, CD3; magenta, CD45; blue, nuclear stain; circles, regions of interest chosen for subsequent gene-expression analysis.

Haematoxylin and eosin (H+E) stained slides were viewed by a pathologist (MW) and suitable areas for analysis were identified, including areas of normal-appearing mucosa and inflamed tissue for each patient, and areas of cancer and/or dysplasia where present (**Figure 1**). Tissue sections (5µm) were cut from corresponding formalin-fixed paraffin-embedded (FFPE) blocks and areas of interest macro-dissected and mounted on Superfrost slides (Thermo Fisher). Spatial profiling was undertaken on the NanoString GeoMx™ Digital Spatial Profiling platform at the Central Facility for Genomics at Griffith University (Gold Coast, Australia). Briefly, tissue sections were stained with Pan-cytokeratin (PanCK), CD45 and CD3 and a nuclear stain and visualisation of the stained slides facilitated selection of regions of interest (ROIs) for subsequent gene expression. ROIs were chosen by the pathologist to contain approximately 300 cells, as defined by the nuclear marker, and approximately equal numbers of PanCK, CD45 and CD3-staining cells. Areas containing fibrosis or necrosis were not chosen. **Figure 1** shows a representative H+E stained section of colorectal cancer tissue and corresponding morphology-stained section, with ROIs chosen for subsequent gene expression analysis marked with circles. Within these selected ROIs, gene expression of 1,812 targets was assessed using the NanoString Cancer Transcriptome Atlas. One hundred and eight non-segmented ROIs and 26 ROIs segmented by PanCK, CD45 and CD3 were selected for gene expression analysis (**Supplementary Table 1** for breakdown of ROIs). Samples were sequenced on a NextSeq 2000 and sequence data processed with the GeoMx DSP software.

#### Digital spatial profiling analysis

RNA profiling was undertaken using the NanoString CTA that includes 1,834 protein-coding human genes based on the human gene nomenclature committee (HUGO) database cross-referenced with available mRNA sequences in the National Centre for Biotechnology’s Information (NCBI) RefSeq database (**Figure 1**), 32 reference genes for normalisation and 75 negative control targets. Samples were sequenced on an Illumina NextSeq, and sequence data processed with the GeoMx DSP software.

### Data analysis

Raw counts were exported from GeoMx DSP for downstream analysis in R. Three regions of interest with very low total counts were removed from analysis. Counts were summarized to the gene level by summing passed probes for each gene, using a filtering method similar to ‘Biological Probe QC’ as described in the GeoMX NGS Data analysis user manual (v2.0). Specifically, if the geomean of a probe across all samples is less than 10% of the sum for all probes for a given gene (low counts) it is excluded. Or, if it fails a Grubbs outlier test (one sided up or down, p-value <0.01) in more than 20% of samples. No gene-level filtering was performed.

Gene counts were normalised with RUVIII, using the 100 genes with minimum calculated variance (v9.7.1, k=5) (17). To minimise potential issues with different cellular composition, the non-segmented ROI differential expression analyses used a normalisation among ‘non-segmented’ regions only, whereas the morphology region analyses (and all other analyses) used values normalised on all passed regions. Principal components were calculated on RUVIII normalised data (removing 10% of low-variance genes).

Differential expression was calculated on normalised values using limma (v3.48), which uses a moderated t-test for statistical analyses (18). Comparative analyses were blocked on individual/slide, with inter-duplicate correlation for pseudo-biological replicate ROIs (multiple samplings from individuals).

Calculated hypergeometric enrichment of significantly differentially expressed genes against NanoString’s provided annotations, KEGG and GO Biological Processes, using a background of panel genes (and only those with any annotations for KEGG or GO) with the hypeR package (v1.8.0) (19).

Cell type composition within whole ROIs was estimated with the SpatialDecon (v1.2.0) (20), package using the safeTME reference dataset (with merged cell types) with a reduced threshold (lower_thresh = 0.01).

## Results

### Patient selection and samples

Sixteen patients with a confirmed diagnosis of IBD were identified from the databases and gave consent for the use of their tissue. The median age was 59 years at the time of resection (range 23-75 years); 11 were male (69%). Of these, ten (63%) had a diagnosis of UC, five (31%) had Crohn’s disease and 1 (6%) had indeterminate colitis. Nine patients had CAC, two had dysplasia only and five had colitis only. One patient had dysplasia in addition to CAC (see **Table 1**). Histologically normal tissue was available for all patients, inflamed tissue from 13 patients, dysplastic tissue from three patients and cancerous tissue from nine patients (see **Supplementary Table S1**).

**Table 1:** Demographic and disease details of included patients.

ID	UC/IC/CD	Worst Dysplasia	Site	Duration of Exposure	Extent of Inflammation	Tissue available
1	IC	No	NA	12 y	Extensive	N, Inf
2	UC	No	NA	34 y	Pancolitis	N, Inf
3	UC	T3N0M0	Rectum	41 y	Pancolitis	N, CAC
4	CD	LGD	Tx	4 y	Right & Tx	N, Inf, D
5	UC	T2N0M1a	Right	14 y	Proctitis	N, CAC
6	UC	No	NA	NK	Pancolitis	N, Inf
7	UC	T2N0M0	Rectum	NK	Left	N, Inf, CAC
8	CD	T3N0M0	Rectum	25 y	Pancolitis	N, Inf, D, CAC
9	CD	No	NA	NK	Extensive	N, Inf
10	UC	T4N0M0	Sigmoid	16 y	Left	N, Inf, CAC
11	UC	T1N0M0	Rectum	11 y	Left	N, Inf, CAC
12	UC	LGD	Rectum	2 y	Proctitis	N, Inf, D
13	CD	T4N2M0	Right	9 y	Pancolitis	N, Inf, CAC
14	UC	T4N2M0	Rectum	NK	Pancolitis	N, CAC
15	UC	No	NA	2 y	Left	N, Inf
16	CD	T3N0M0	Rectum	25 y	Left	N, Inf, CAC

(ID, identification; UC, ulcerative colitis; IC, indeterminate colitis; CD, Crohn’s Disease; M, male; F, female; LGD, low grade dysplasia; NA, not applicable; y, years; NK, not known; Tx, transverse); N, normal; Inf, inflamed; D, dysplasia; CAC, colitis-associated colorectal cancer.

### Comparison of tissue type by gene expression

**Figure 2:**
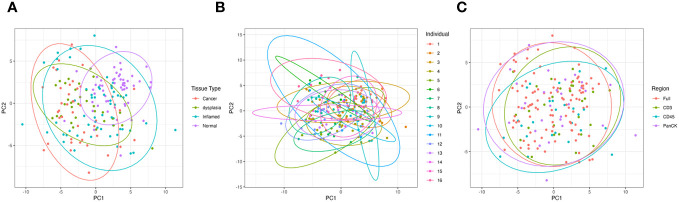
Principal Component Analysis (PCA) of all samples based on gene expression showing the first two principal components. **(A)** is grouped by tissue type, **(B)** grouped by individual patient and **(C)** grouped by morphology regions.

Tissue type is the greatest driver of sample clustering based on gene expression; plotting the first two principal components of each sample shows clustering by tissue group (**Figure 2A**), and does not appear to be overly driven by individual (**Figure 2B**) or morphology marker (**Figure 2C**).

**Figure 3:**
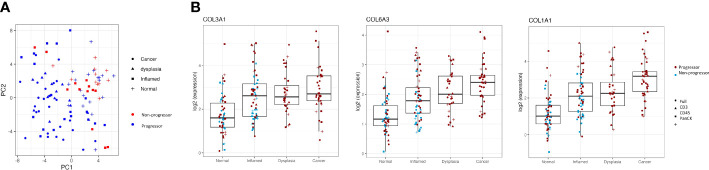
Clustering of progressors and non-progressors is largely due to differences in gene expression of collagen genes. **(A)** shows a clear separation between progressors and non-progressors, as seen in the PC1 dimension. Red symbols denote non-progressors (IBD patients); blue symbols denote progressors (dysplasia/cancer patients). **(B)** shows expression of collagen genes (*COL1A1, COL3A1* and *COL6A3*) in all regions of interest from the different tissue types (normal, inflamed, dysplastic and cancer). Differential gene expression between each of the tissue types is statistically significant (adjusted P values all <0.003) for all three genes.

Interestingly, there is separation between the individuals with cancer/dysplasia vs those with IBD alone seen in the PC1 dimension (**Figure 3A**). However, this is largely driven by high weightings of three collagen genes; *COL1A1, COL3A1* and *COL6A3*, which steadily increase in inflamed, dysplasia and cancer samples compared to normal tissue samples, with adjusted P-values <0.003 (**Figure 3B**).

### Differences in molecular programmes are seen between tissue types

**Figure 4:**
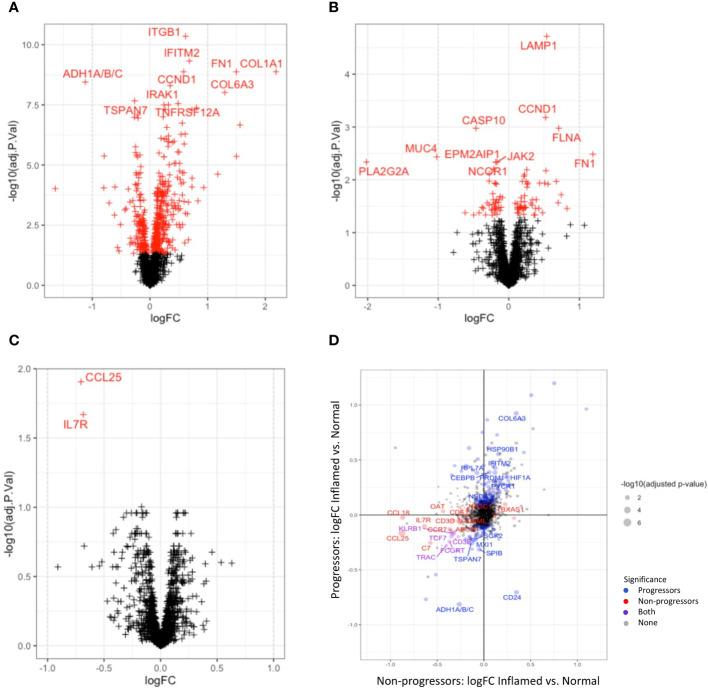
Differential gene expression between tissue types. Volcano plots illustrating differentially expressed genes between cancer and normal tissue **(A)** and cancer and dysplastic tissue **(B)**. Red crosses indicate significant adjusted P-values; crosses on the right are overexpressed in cancer. **(C)** shows the two genes (IL17R and CCL25) that are significantly over-expressed in normal tissue of non-progressors compared to normal tissue of progressors. **(D)** plots the differential gene expression between inflamed and normal tissue of progressors vs non-progressors.

Tissue group-wise differential expression analyses show substantial significant differences when comparing full ROIs, indicating reprogramming at the molecular level. Areas of cancer tissue had significantly more highly expressed genes involved in matrix remodelling, including *COL1A1, COL1A2, COL3A1, COL6A3, CEACAM6, FN1* and many genes previously reported to be over-expressed in cancer, including *CCND1, SPP1, MYC, CTNNB1* and *RHOB*, when compared to histologically normal tissue and inflamed tissue (**Figure 4A**). In addition, genes reported to be involved in immunological response to pathogens, such as *IL-8, IFITM1, IFITM2*, and *RHOB* were also significantly differentially expressed in cancer tissue. When comparing cancer to dysplastic tissue, there were fewer significantly over-expressed genes, but these also included genes involved in extra-cellular matrix remodelling (*COL1A1, COL1A2, CEACAM6, ACTA2*) and carcinogenesis (*MYC, CTNNB1, CCND1*), indicating a progression of cellular remodelling from normal through dysplastic to cancer tissue (**Figure 4B**).

Significantly down-regulated genes in cancer tissue, compared to normal tissue included *PLA2G2A, MUC4, PCK1, TXNIP*, and genes encoding the CCR7 ligands, *CCL19* and *CCL21*, which are lymphoid chemokines involved in the chemotaxis of lymphoid cells such as leukocytes and dendritic cells. Together with significantly lower expression of *CD24, CD79A, CD27, IL7R* and *CD3E* in cancer tissue, these findings suggest dysregulated innate and adaptive immune responses in cancer tissue **(Supplementary Tables** with gene lists).

Comparison of dysplastic areas of tissue to normal and inflamed areas show similar changes to cancer tissue. In addition, over-expression of genes such as *CXCL1*, 2 and 3, and *LCN2* and *RHOB* suggest an immunological response to pathogens as part of the dysplastic process.

**Figure 5:**
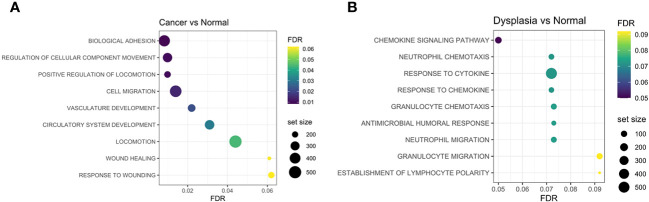
Gene-set enrichment analyses identified adhesion processes, cell migration, vasculature development and wound-healing response as being more enriched in cancer samples compared to normal **(A)**, while chemokine and cytokine activation and humoral responses are enriched in dysplastic samples compared to normal tissue **(B)**. FDR, false discovery rate.

Gene-set enrichment analysis (GSEA) highlights the different molecular mechanisms at play in the different tissue groups. Enrichment of cell-adhesion, matrix remodelling and epithelial-mesenchymal transition (wound healing) gene sets was seen in cancer tissue (**Figure 5A**), while dysplastic tissue was characterised by anti-microbial humoral immune response and response to cytokines, when compared to normal tissue (**Figure 5B**).

### Differences in molecular programmes are seen between progressors and non-progressors

Disease-wise differential expression analyses that compare IBD patients with dysplasia/cancer (progressors) to those without (non-progressors), show significant differences when comparing full ROIs, indicating molecular changes associated with development of dysplasia and CAC in the context of IBD. Differential gene expression between progressors and non-progressors showed a decrease in expression of genes involved with both innate and adaptive immune processes, e.g. CCL25, IL7R, CCL19, and CCL21, in both inflamed and normal tissue, although these differences were not always significant after correction for multiple testing (**Figure 4C**). Collagen-coding genes were also overexpressed in both normal and inflamed tissue between disease types. In order to more closely analyse genes that change differently between normal and inflamed tissues in progressors and non-progressors, we compared differential expression results between the inflamed-normal analyses within progressors, to the inflamed-normal analyses non-progressors. Notable findings are an decrease in the oncogene, CD24, in inflamed tissue compared to normal tissue of progressors, which is not seen in non-progressors. In contrast, a decrease in CCL18, CD3 and CD6 is seen in inflamed vs. normal tissue of non-progressors compared to progressors (**Figure 4D**).

**Figure 6:**
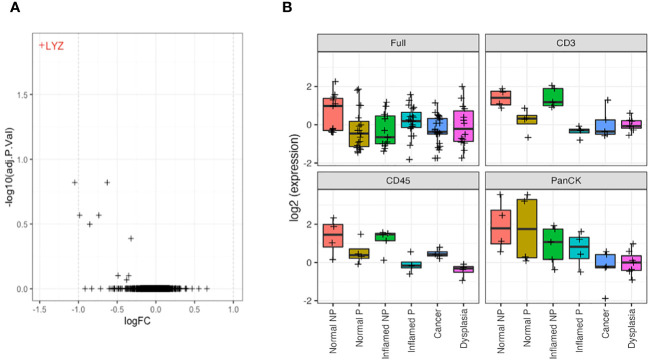
*LYZ*, encoding lysozyme, is overexpressed in non-progressors. **(A)**. Differential gene expression between progressors (right) and non-progressors (left) in CD3-positive regions of interest. *LYZ*, encoding lysozyme, was the only gene significantly over-expressed in non-progressors. **(B)**. Lysozyme gene expression (LYZ) across each tissue type from full regions of interest, CD3-positive cells, CD45-positive cells and PanCK-positive regions. NP, non-progressor; P, progressor.

Morphology region comparisons (delineated by CD3, CD45 or PanCK+ within ROI) had less overall significant differential expression due to smaller sample numbers. However, where a gene is significantly differentially expressed in a non-segmented ROI (after multiple hypothesis correction), it can be appropriate to consider its raw uncorrected p-value within a morphology region, as its difference is already established. The LYZ gene, coding for lysozyme, was significantly decreased in CD3-positive morphology regions of all types of tissue in IBD patients with dysplasia/CAC (progressors), compared to those without dysplasia/CAC (**Figure 6A**).

Further analysis of *LYZ* gene expression showed a significant increase in normal tissue of patients with IBD only, compared to any tissue type from patients who also had dysplasia/CAC, and inflamed tissue from IBD patients, when examining full ROIs. Analysis of expression by morphology region showed a decreased/absent expression of *LYZ* in CD3 and CD45-positive cells from patients with dysplasia/CRC in contrast to the significant elevation of expression in tissue from patients with IBD alone (logFC = 1.6; *P* = 0.001). This suggests an underlying mechanism of neoplastic disease progression involving dysregulated lysozyme production, and may represent a potential predictive biomarker of dysplasia/CRC development in IBD patients (**Figure 6B**).

### Cell deconvolution

**Figure 7:**
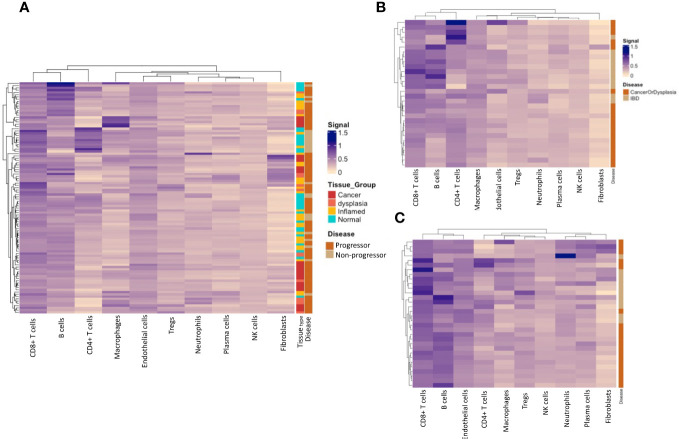
Immune-cell deconvolution grouped by tissue type and progressor/non-progressor. **(A)**. Immune-cell deconvolution using gene expression from full regions of interest demonstrates heterogeneity between samples and identifies potential disease subtypes characterised by specific immune-cell infiltrate. **(B)** shows that a subset of progressors display a concentration of B-cells in their normal tissue, while a subset of non-progressors are characterised by CD4+ T-cells in their normal tissue. No notable clustering of immune-cell populations was seen in inflamed tissue **(C)**.

We calculated cell type-specific signals using the spatialDecon package, using the ‘safe-TME’ matrix intended for solid tumour samples. These are presented on heatmaps, clustered by cell-type signal. Cell types with low signal (average ROI signal < 0.1; monocytes, pDC, mast and mDCs), were omitted from the heatmaps. Analysis of full ROIs showed several cell-type signal trends (**Figure 7A**), including a high macrophage signal in a subset of cancer tissue samples, and the highest CD4+ T-cell signal in a subset of normal/inflamed tissues. A grouping of higher fibroblast signal is seen in a subset of cancer/dysplasia samples, and excludes normal tissue, which might be driven by the collagen gene COL1A1, which has the highest fibroblast weighting in the reference matrix. Cell-type deconvolution using data from normal tissue samples only (**Figure 7B**) shows a concentration of CD4+ T cell signal signature within those with dysplasia and/or CAC and B cells within IBD samples. No such trends are seen when analysing deconvoluted data from the inflamed tissue samples (**Figure 7C**).

## Discussion

Recent studies have shown that the organisation and structure of cancer cells within a tumour, known as tumour microenvironment, plays a significant role in the development and progression of cancer. This includes the spatial distribution of different cell types within the tumour, as well as the formation of specific structures such as blood vessels and immune cells. Understanding the tumour architecture can provide important insights into the biology of a cancer, and may help to improve diagnosis, treatment, and prognosis. Previous research techniques have described quantitative differences in protein and gene expression in colitis-associated CRC (CAC), however, most have lacked the capacity to localise the site of expression, limiting the interpretation of this data. Novel spatial profiling techniques, such as Digital Spatial Profiling (DSP), allow a more nuanced insight into the alterations of gene expression and dysregulation of cellular pathways in CAC, which is critical to the development of clinically relevant biomarkers for prognostication in IBD. This study identified significant differences in gene expression as well as immune-cell infiltration between normal, inflamed, dysplastic and CAC tissue. Additionally, the normal and inflamed tissue of progressors and non-progressors show significantly different gene expression.

Unsurprisingly, there were several genes that were differentially expressed across tissue types. The most marked cluster comprised of collagen genes associated with tissue remodelling (*COL1A1*, *COL3A1* and *COL6A3*); these show steady increase in expression across normal, inflamed, dysplastic and cancer tissue. This is consistent with existing literature in non-IBD CRC, and demonstrates the integral role of the collagen in the tumour microenvironment, including interfacing with the immune population and promoting metastasis (21, 22).

Overexpression of the proto-oncogene, *MYC*, was observed in dysplastic and cancer tissue, similar to a study of 47 CAC patients, where a higher rate of *MYC* amplification was reported compared to sporadic CRC, highlighting its importance to the CAC pathway, specifically (23). *MYC* is a transcription factor responsible for modulating cellular proliferation, and orchestrating changes in the tumour microenvironment, including angiogenesis and immune response. *MYC* is a key factor in Wnt pathway activation, which is an early event in CRC development. While in sporadic CRC, the Wnt pathway is most commonly activated through mutations in the *APC* gene, these mutations are seen less frequently and later in CAC. Overexpression of *MYC* suggests a mechanism by which the Wnt pathway may be activated in the absence of a primary *APC* mutation, with a similar oncogenic effect.

The role of mucins and the significance of changes in mucin gene expression has been controversial in both IBD and CRC (24, 25). *MUC4* codes for one of the mucins contributing to the protective mucous layer lining the small and large bowel, and its over-expression has been associated with both poorer outcome (26) and enhancement of anti-tumour response (27). *MUC4* ^-/-^ mice showed an attenuated colitic response to dextran sodium sulphate and a reduced tumour burden compared to wild-type mice suggesting a role for *MUC4* in inflammation and progression to neoplasia, however, the discrepancy between studies implies it is a complex relationship (28). We found a significantly lower expression of *MUC4* in dysplastic and cancer tissue, suggesting that progressive loss of *MUC4* may be implicated in the development of CAC.

In order to identify potential early biomarkers of disease progression, a comparison of normal and inflamed tissue from ‘progressors’ and ‘non-progressors’ was performed. The *LYZ* gene, coding for lysozyme, an enzyme produced to digest bacterial cell walls, was dramatically under expressed in the progressors in contrast to the non-progressors, and this under-expression was driven by the immune-cell components in the tissue microenvironment. Lysozyme is normally produced by the Paneth cells of the healthy caecum and right-sided colon. In IBD, Paneth cell metaplasia is seen in the remaining colon with an associated increase in lysozyme production (29). Both faecal and serum lysozyme have been proposed as markers of disease severity in IBD (30). More recently, a series of murine experiments have demonstrated an exaggerated colitic response associated with the ectopic expression of lysozyme; in addition, the authors showed that loss of *LYZ* expression affected the immune populations through its effect on the mucosal microbiome (31).

The critical role of lysozyme in the regulation of the microbiome may provide a mechanism through which it may drive chronic inflammation and neoplastic transformation. Lysozyme has been shown to have inhibitory effects on tumour proliferation through both direct and indirect modulation of host immunity, inducing interferon production, as well as interruption of both interleukin and S100A6 signalling. *In vitro* co-culture with lysozyme has led to inhibition of proliferation in a variety of cancer cell lines (32, 33). This suggests that the loss of expression impairs mucosal immunity and promotes tumour development, and highlights the potential use of *LYZ* as a biomarker of progression to CAC.

The oncogene, CD24, was consistently over-expressed in progressors compared to non-progressors in this study. It is a small cell-surface glycoprotein, expressed on a number of immune cells, that plays a role in both signal transduction, immune regulation, and cellular adhesion. It has been implicated in early-stage carcinogenesis, as well as poorer prognosis tumours (34). In the APC^Min/+^ mouse model of colorectal carcinogenesis, CD24 knock-out reduces tumour development in a dose-dependent way (reduction in burden with heterozygous loss and no tumour production with homozygous knock-out) (35). This is a promising biomarker to discriminate progressors from non-progressors and even has potential as a therapeutic target; antibodies to CD24 have been used in in *in vitro* and murine models to suppress invasion and proliferation of tumour cells through interruption of STAT3 phosphorylation (36), and Ras/BCL-2 pathway down-regulation (37, 38).

The importance of the tumour immune microenvironment is of particular significance to CAC, given the proposed role of chronic inflammation in the development of neoplasia. Analysis of immune cell populations suggests significant heterogeneity, with clustering of different possible sub-types within the CAC group. It is likely that this represents the differences in immune activation and CAC may in fact encompass a diverse collection of distinct CRC pathways and phenotypes. Similarly, a recent single-cell RNA-sequencing and spatial transcriptomics study by Garrido-Trigo et al. identified macrophage and neutrophil components to have the highest heterogeneity among IBD patients (39). Although this study did not include patients with progressive disease, diversity in immune and stromal cell components points to patient-dependent factors that may explain the heterogeneity seen in IBD and be important for progression to CAC. We also identified several genes associated with immune activity that were differentially expressed between progressors and non-progressors, including *CCL25*. In normal, non-inflamed tissue, the chemokine CCL25 is expressed in the small bowel and not typically in the colon and plays a role in T- and B-cell recruitment. CCL25 expression has been shown to correlate with the presence and severity of colitis (40). In our study, progressors showed a reduced expression of *CCL25* compared to those that did not progress, suggesting a difference in lymphocyte recruitment in the development of CAC, compared to patients with IBD only. Similarly, IL-7 receptor (*IL-7R*) and *CCL18* gene expression was also reduced in progressors compared to non-progressors. Higher levels of expression of IL-7R are associated with non-responsiveness to anti-TNF therapy in IBD and hence, higher inflammatory burden (36), while *CCL18* is a chemokine that induces differentiation of T-cells towards a ‘pro-tumour’ type macrophage and facilitates matrix remodelling and immune evasion (41, 42). While the reduced expression of these pro-inflammatory mediators may reflect the higher inflammatory burden in the study population undergoing colectomy for medically refractory colitis, compared to the patient group who underwent colectomy for dysplasia or cancer, it may also represent underlying differences in immune activation between the two patient groups.

The T-cell markers, CD3 and CD6, showed lower gene expression in the normal and inflamed tissue from progressors compared to non-progressors. Previous immune profiling studies have shown a reduction in CD3^+^ and CD8^+^ cells in CAC compared to sporadic CRC (43). In contrast, a study comparing microsatellite stable CAC and sporadic CRC demonstrated an increase in CD3^+^ and CD8^+^ cells in CAC, but without an associated increase in tumour cell apoptosis (44). Our study uses different comparators both in terms of the tissue used (normal/inflamed), and the comparator groups (progressors/non-progressors), making it difficult to directly compare the data. However, it is likely that inherent differences in the immune populations and their function contribute to the risk of progression.

The major limitation of this feasibility study is the retrospective nature and limited numbers of patients available. Use of colectomy specimens has meant that there is an inherent difference in the population groups due to the different indications for surgery. This has been compensated for to some degree with the use of histologically normal tissue from the different patients. While this study has demonstrated the potential of DSP to identify biomarkers from a small cohort and the importance of the spatial context of biomarker discovery, validation studies are necessary to confirm the preliminary findings from this small cohort.

This study has demonstrated key differences in gene expression across tissue types and between progressors and non-progressors. The future potential of this study findings is in the identification and confirmation of early changes that predict progression to dysplasia and cancer, enabling better risk stratification, more accurately informed clinical decision making and future therapeutic target development. Future studies should focus on prospective validation of these findings to confirm and better develop understanding of these findings. This is an area of clinical need, where an accurate biomarker would have the potential to revolutionise clinical management for these patients.

## Data availability statement

The authors acknowledge that the data presented in this study must be deposited and made publicly available in an acceptable repository, prior to publication. Frontiers cannot accept a article that does not adhere to our open data policies.

## Ethics statement

The studies involving humans were approved by Health and disability ethics committees of New Zealand. The studies were conducted in accordance with the local legislation and institutional requirements. The participants provided their written informed consent to participate in this study.

## Author contributions

TG was involved in study design, patient selection, data acquisition, manuscript writing and editing. SW was responsible for data analyses and bioinformatics MW was responsible for histopathological assessment and sample selection TE was involved in study design and manuscript writing and editing NW was involved in study design, methodology and data analyses RP was involved in study design, data analyses, manuscript writing and editing
